# HIV self-testing among female sex workers in Zambia: A cluster randomized controlled trial

**DOI:** 10.1371/journal.pmed.1002442

**Published:** 2017-11-21

**Authors:** Michael M. Chanda, Katrina F. Ortblad, Magdalene Mwale, Steven Chongo, Catherine Kanchele, Nyambe Kamungoma, Andrew Fullem, Caitlin Dunn, Leah G. Barresi, Guy Harling, Till Bärnighausen, Catherine E. Oldenburg

**Affiliations:** 1 John Snow, Inc., Lusaka, Zambia; 2 Department of Global Health and Population, Harvard T.H. Chan School of Public Health, Boston, Massachusetts, United States of America; 3 John Snow, Inc., Boston, Massachusetts, United States of America; 4 Department of Epidemiology, Harvard T.H. Chan School of Public Health, Boston, Massachusetts, United States of America; 5 Research Department of Infection and Population Health, Institute for Global Health, University College London, London, United Kingdom; 6 Heidelberg Institute of Public Health, University of Heidelberg, Heidelberg, Germany; 7 Africa Health Research Institute, KwaZulu-Natal, South Africa; 8 Francis I. Proctor Foundation for Research in Ophthalmology, University of California, San Francisco, California, United States of America; 9 Department of Ophthalmology, University of California, San Francisco, California, United States of America; 10 Department of Epidemiology and Biostatistics, University of California, San Francisco, California, United States of America; Desmond Tutu HIV Centre, SOUTH AFRICA

## Abstract

**Background:**

HIV self-testing (HIVST) may play a role in addressing gaps in HIV testing coverage and as an entry point for HIV prevention services. We conducted a cluster randomized trial of 2 HIVST distribution mechanisms compared to the standard of care among female sex workers (FSWs) in Zambia.

**Methods and findings:**

Trained peer educators in Kapiri Mposhi, Chirundu, and Livingstone, Zambia, each recruited 6 FSW participants. Peer educator–FSW groups were randomized to 1 of 3 arms: (1) delivery (direct distribution of an oral HIVST from the peer educator), (2) coupon (a coupon for collection of an oral HIVST from a health clinic/pharmacy), or (3) standard-of-care HIV testing. Participants in the 2 HIVST arms received 2 kits: 1 at baseline and 1 at 10 weeks. The primary outcome was any self-reported HIV testing in the past month at the 1- and 4-month visits, as HIVST can replace other types of HIV testing. Secondary outcomes included linkage to care, HIVST use in the HIVST arms, and adverse events. Participants completed questionnaires at 1 and 4 months following peer educator interventions. In all, 965 participants were enrolled between September 16 and October 12, 2016 (delivery, *N =* 316; coupon, *N =* 329; standard of care, *N =* 320); 20% had never tested for HIV. Overall HIV testing at 1 month was 94.9% in the delivery arm, 84.4% in the coupon arm, and 88.5% in the standard-of-care arm (delivery versus standard of care risk ratio [RR] = 1.07, 95% CI 0.99–1.15, *P =* 0.10; coupon versus standard of care RR = 0.95, 95% CI 0.86–1.05, *P =* 0.29; delivery versus coupon RR = 1.13, 95% CI 1.04–1.22, *P =* 0.005). Four-month rates were 84.1% for the delivery arm, 79.8% for the coupon arm, and 75.1% for the standard-of-care arm (delivery versus standard of care RR = 1.11, 95% CI 0.98–1.27, *P =* 0.11; coupon versus standard of care RR = 1.06, 95% CI 0.92–1.22, *P =* 0.42; delivery versus coupon RR = 1.05, 95% CI 0.94–1.18, *P =* 0.40). At 1 month, the majority of HIV tests were self-tests (88.4%). HIV self-test use was higher in the delivery arm compared to the coupon arm (RR = 1.14, 95% CI 1.05–1.23, *P =* 0.001) at 1 month, but there was no difference at 4 months. Among participants reporting a positive HIV test at 1 (*N =* 144) and 4 months (*N =* 235), linkage to care was non-significantly lower in the 2 HIVST arms compared to the standard-of-care arm. There were 4 instances of intimate partner violence related to study participation, 3 of which were related to HIV self-test use. Limitations include the self-reported nature of study outcomes and overall high uptake of HIV testing.

**Conclusions:**

In this study among FSWs in Zambia, we found that HIVST was acceptable and accessible. However, HIVST may not substantially increase HIV cascade progression in contexts where overall testing and linkage are already high.

**Trial registration:**

ClinicalTrials.gov NCT02827240

## Abstract 2

In a cluster-randomized trial done in Zambia, Catherine Oldenburg and colleagues study HIV self-testing for female sex workers.

## Author summary

**Why was this study done?:**

- HIV self-testing can be done in the absence of a health provider and may close gaps in the HIV treatment and prevention cascades.
- Female sex workers are at increased risk of HIV acquisition and may particularly benefit from HIV self-testing because it could address some of their specific barriers to HIV testing, e.g., health provider stigma and discrimination, health facility hours, and distance to the health facility.
- How HIV self-tests are delivered to female sex workers may impact the effectiveness of this HIV testing intervention.

**What did the researchers do and find?:**

- We measured the effectiveness of 2 HIV self-testing distribution mechanisms by randomizing 965 female sex workers in 3 Zambian transit towns to (1) direct delivery of an HIV self-test (delivery arm), (2) a coupon for collection of an HIV self-test from a health clinic/pharmacy (coupon arm), and (3) referral to standard-of-care HIV testing and counseling (standard-of-care arm) (all provided by peer educators).
- At the 1-month and 4-month follow-up, overall HIV testing in the previous month was high (1-month: 94.9% delivery arm, 84.4% coupon arm, 88.5% standard-of-care arm), and there were few statistically significant differences across study arms.
- Among participants reporting an HIV-positive test result, linkage to HIV-related care and ART initiation were non-significantly lower among those in the HIV self-testing arms compared to the standard-of-care arm at both time points, although there was limited power to detect differences.
- Three adverse events related to HIV self-testing were reported over the duration of the study, all of which were intimate partner violence.

**What do these findings mean?:**

- HIV self-testing appears safe, acceptable, and accessible among female sex workers in Zambian transit towns.
- HIV self-testing, regardless of the delivery model, may not sustainably increase HIV cascade progression in contexts where overall HIV testing is high.

## Introduction

Achieving high HIV testing coverage is essential for realizing the first step of the Joint United Nations Programme on HIV/AIDS (UNAIDS) 90-90-90 target of diagnosing 90% of all people living with HIV by 2020 [1]. In December 2016, the World Health Organization (WHO) released guidelines related to HIV self-testing (HIVST) [2,3], recommending that HIVST be offered in addition to standard HIV testing services to help achieve realization of this target and as an entry point into HIV prevention services for those testing negative. In particular, the guidelines recognize the importance of the development of new approaches such as HIVST for members of key populations that frequently have lower uptake of HIV testing services due to multilevel factors such as healthcare provider stigma [4,5] and lack of legal protection [6].

Oral HIVST has been shown to be acceptable in diverse populations globally, and provision of HIV self-tests has been shown to increase HIV testing compared to standard testing services in some populations [7–10]. Currently, limited data exist regarding HIVST among female sex workers (FSWs). A concurrent randomized controlled trial of HIVST among FSWs in Kampala, Uganda, found that HIVST increased recent and repeat HIV testing compared to referral to standard testing services [11]. A cohort study among FSWs in Kenya found that 71% of participants used an HIV self-test after it was made available to them, but this study did not include a comparison group for standard testing services [12]. WHO recommends frequent retesting for members of key populations, including FSWs [3]. Although there are limited data on the HIV care continuum for FSWs, available estimates suggest that all indicators are far behind the 90-90-90 targets [13–15]. Alternative testing strategies, such as HIVST, may help close the gap between current HIV testing coverage among FSWs and achieving UNAIDS’s first 90% target of diagnosing 90% of all people living with HIV by 2020.

Even though HIVST may reduce some barriers to HIV testing, low access to or uptake of HIVST would limit its ability to improve HIV testing coverage. Here, we test 2 HIVST delivery mechanisms—direct delivery of an HIV self-test and facility-based distribution—compared to standard-of-care HIV testing among transit-town-based FSWs in Zambia. The 2 HIVST arms were designed to evaluate whether HIVST is acceptable (by measuring whether individuals who are directly given the test use it) and whether participants will access HIVST via the existing health system (by measuring whether participants collect and use HIV self-tests from existing facilities) [16]. The second arm is important because it mirrors the likely approach countries will take in providing routine access to HIVST. We hypothesized that the active approach of peer-based HIV self-test delivery would perform better in terms of HIV testing and HIV status knowledge than the more passive facility-based approach. We further hypothesized that both types of HIV self-test kit provision would lead to significantly improved recent HIV testing and better HIV status knowledge compared to standard testing.

## Methods

### Study design

The Zambian Peer Educators for HIV Self-Testing (ZEST) study was a 3-arm 1:1:1 cluster randomized trial evaluating the effect of 2 different health system mechanisms for HIV self-test delivery compared to referral to standard HIV testing. Clusters were defined as groups of FSW participants and a peer educator who recruited them and facilitated interventions. Complete methods for the ZEST study have been previously reported [16]. The full trial protocol is available online (S1 Protocol). Institutional review board approval was obtained from the Harvard T.H. Chan School of Public Health in Boston, MA, and the ERES Converge institutional review board in Lusaka, Zambia. Written informed consent was obtained from all participants.

### Peer educators

Participants were recruited in 3 Zambian transit towns (Kapiri Mposhi, Chirundu, and Livingstone) by peer educators. Peer educators were current or former FSWs who had been recruited and trained by study staff prior to study initiation; many had formally worked as peer educators for previous FSW implementation projects in their region. Peer educators were recruited via contacts with current or former FSW organizations in each study town by study staff members. Peer educators were hired based on their willingness to participate for the duration of the study and their reliability. All peer educators were 18 years of age or older and self-reported being current or former sex workers [17]. There was no enumeration list or sampling frame of FSWs in the study community. Thus, peer educators recruited members of their social network via direct contact, and referred interested individuals to study staff for eligibility assessment and enrollment. We purposefully chose this sampling approach because we wanted to carry out our test of the effect of alternative delivery strategies for HIVST among FSWs who could be easily reached through peer networks. This sampling approach allows similar HIVST interventions to be carried out in other settings.

### Participants and procedures

Potential participants contacted a research assistant by phone for preliminary eligibility screening and then, if eligible, were formally screened and enrolled in person. Eligible participants were 18 years of age or older at the time of enrollment, had exchanged sex (vaginal, oral, and/or anal) for money or goods at least once in the past month, self-reported an HIV-uninfected status and had not had an HIV test in the previous 3 months or self-reported that their HIV status was unknown, and were permanent residents of their study town of enrollment. The target enrollment was 6 study participants per peer educator.

### Randomization

Peer educator–participant groups were randomized as clusters in a 1:1:1 fashion to 1 of the 3 study arms: (1) direct delivery of the HIV self-test from the peer educator to the participant (henceforth, delivery), (2) distribution of a coupon from the peer educator to the participant that could be used for collection of an HIV self-test from a fixed distribution point (henceforth, coupon), or (3) referral to standard testing (henceforth, standard of care). Group randomization occurred after all of the participants in a group had completed their baseline study assessment. The randomization list was generated in R (version 3.3.1, R Foundation for Statistical Computing, Vienna, Austria) in random blocks of size 3, 6, and 9 and stratified by study site (Kapiri Mposhi, Chirundu, or Livingstone) to ensure balance in study arms by site. Because of the nature of the intervention, the study was not masked; however, the peer educator’s study arm assignment was concealed until all participants in her group had been enrolled.

### Interventions

In all study arms after randomization, participants completed 4 peer educator intervention visits at weeks 0, 2, 6, and 10 that consisted of HIV risk reduction counseling, condom distribution, and information on where to get HIV testing. The first intervention visit was conducted in a group, and all subsequent interventions were one-on-one visits between the peer educator and participant. To emulate real-life peer educator interventions and improve the generalizability of our results, study staff were not present at peer educator visits. Participants reported how many times they met with their peer educator in the past month at each study assessment.

In the delivery arm, peer educators distributed 2 HIV self-test kits (OraQuick ADVANCE Rapid HIV-1/2 Antibody Test, OraSure Technologies, Bethlehem, PA): one at the first (week 0) peer educator intervention visit, and a second one at the fourth (week 10) peer educator intervention visit. The kits included the manufacturer’s pictorial and written instructions in English, Nyanja, Bemba, and Tonga. Peer educators were trained on use of the oral HIV self-test and shared this information with participants. All participants in the HIVST arms were offered a second HIV self-test kit, regardless of HIV status. To preserve participant confidentiality, peer educators did not ask participants their HIV status. The second HIV self-test kit distribution was timed to measure repeat use of HIVST by participants. We distributed the second test kit at an interval approximating the manufacturer’s recommended testing interval for repeat HIVST.

In the coupon arm, peer educators distributed coupons that participants could use to collect an OraQuick HIV self-test at a distribution site, which was an existing health facility (health clinic or pharmacy). HIV self-test kits were distributed free of charge to participants in exchange for the coupon. HIV self-tests were not available for purchase to participants in other study arms. There was no change in the health facilities with regards to hours of operation or staffing. Existing staff were briefly trained on study procedures and the use of the HIV self-test. As with the delivery arm, peer educators distributed 1 coupon at the first (week 0) and fourth (week 10) peer educator intervention visits. The content of the test and instructions provided to participants were identical to those in the delivery arm. As with the delivery arm, there was no HIV status requirement for distribution of the second coupon. To minimize contamination, only participants who had the study coupon received an HIV self-test kit at the facility.

In the standard-of-care arm, peer educators only provided information about existing HIV testing services, including the locations and working hours, where participants could obtain an HIV test. Identical information was provided to participants in the delivery and coupon arms. HIV care and antiretroviral therapy (ART) were available at some, but not all, of the existing HIV testing services. Participants in any arm who tested positive at a facility that did not provide ART were referred to facilities that provided ART.

A 24-hour hotline was made available to participants in all arms. Participants were instructed to call the hotline if they needed help with HIV testing (including using the HIV self-test), experienced any adverse events, and/or needed other assistance.

### Assessments

Assessments occurred at baseline prior to randomization and at 1 and 4 months after the first peer educator visit. All assessments were conducted by a research assistant using computer-assisted personal interviewing.

At baseline, participants were asked about sociodemographic characteristics (age, literacy, educational attainment, mobile phone ownership, monthly income, and if they had a primary partner—defined as a stable, non-commercial partner, such as a husband or a boyfriend). Participants were asked about their sex work history, including the age at which they started exchanging sex for money, average number of clients per night, and condom use with these clients. Inconsistent condom use with clients was defined as reporting non-condom use with any client. Participants were asked if they had ever had an HIV test, and if they had, the number of months since their last test. Finally, participants were asked if any sexual partner (including both commercial and non-commercial partners) had physically (hit, slapped, punched, pushed, shoved, or done something else to physically harm) or sexually (physically forced to have sex) hurt them in the previous 12 months.

The prespecified primary outcome was past 1-month HIV testing at the 1-month and 4-month study assessments. This outcome was chosen as it is applicable to all study arms (i.e., there was no HIV self-test use in the standard-of-care arm), and to measure the overall impact of HIV self-test access on all types of HIV testing (e.g., there could be changes in clinic-based testing as a result of having an HIVST coupon). Data on past 1-month HIV testing at the 4-month visit was collected to measure repeat HIV testing. Participants who reported testing within the study period at the 1- or 4-month visit were considered to have tested at least once over the course of the study. Participants were asked about recent HIV testing history, including when their last HIV test was, where they received the HIV test (facility versus self-test), their HIV status at their last test, and, among those who reported a positive test, if they sought medical care following their positive test and if they initiated ART. In the delivery and coupon arms, participants were asked if they were offered a test/coupon by the peer educator, if they took the test/coupon, if they collected the kit (in the coupon arm), and if they used the HIV self-test. Whereas we assessed HIV testing specifically in the past month for the overall HIV testing outcome, we measured if participants used an HIV self-test independently of when they used it. At 4 months, participants were additionally asked how many kits in total they used during the study period.

To measure HIV status knowledge, research assistants asked participants to self-report their HIV status at the 4-month visit and then take a rapid test to confirm their status. Participants were told that they would receive a small gift (worth approximately US$1) for correctly self-reporting their best guess of their HIV status; all participants received the gift regardless of whether their reported HIV status matched the self-report. Pre-test and post-test counseling by a research assistant was available to all participants who chose to participate in the HIV status knowledge assessment.

To measure actual use of the HIV self-tests, at the end of the study participants were asked to return any unused HIV self-test kits in exchange for approximately US$1. Participants were not told of this offer prior to their 4-month visit to avoid biasing the study. The first buy-back offer was not made until at least 1 month after the last test kit was distributed in the study, to avoid the possibility that rumors of the study buying back kits could lead to participants choosing not to use them.

### Adverse events

Adverse events were monitored in all study arms throughout the course of the study by research assistants and peer educators and via the study hotline. At each peer educator intervention visit and during study assessments, participants were screened for physical, sexual, or verbal intimate partner violence, unintentional disclosure of HIV status, and self-harm, and were given an opportunity to report any other events.

### Sample size determination

Sample size determination was based on the primary outcome: testing for HIV in the past month at the 1-month visit. Power calculations were performed using methods for cluster randomized trials, with the peer educator–participant group as the randomization unit. Based on previous data from FSWs in Livingstone and Chirundu [18,19], we assumed that 50% of participants would have tested in the previous month in the standard-of-care arm, and assumed 20% loss to follow-up. We estimated that 50 peer educators per arm (150 total) and 6 participants per peer educator (900 total) would yield 89% power to detect a risk ratio (RR) of 1.3 for recent testing, assuming a type I error probability of 0.05 and an intracluster correlation of 0.03. During enrollment, 10 additional peer educators were recruited, yielding a total of 160 peer educators and 965 participants.

### Statistical methods

Our prespecified primary outcome was the proportion of participants reporting testing for HIV in the previous 1 month as measured at the 1-month and 4-month assessments. Models of HIV testing at both time points included all participants. All pairwise comparisons for the 3 study arms were prespecified. Our prespecified analysis was a multilevel mixed-effects logistic regression model to account for clustering by peer educator group and study site. To estimate RRs and accommodate how common most outcomes were, we used a mixed-effects generalized linear model with a Poisson distribution, log link, and robust error term [20], with fixed effects for randomization arm and study site and a random effect for peer educator group. Modeling with a binomial distribution and logit link did not change conclusions (S8 Table). Each time point was modeled separately.

Secondary outcomes were analyzed with an identical model. Analyses of seeking medical care for HIV and ART use were restricted to individuals who reported that their most recent HIV test was positive, a post-randomization characteristic. Use of the HIV self-test kit was compared between the 2 HIVST arms (delivery and coupon). This model was identical to that used for the primary outcome, with the exception that the term for study arm contained only 2 levels (delivery or coupon). A similar model was used for being offered the test kit or coupon and taking the test kit or coupon.

### Sensitivity analyses

As a sensitivity analysis, we calculated the proportion of participants within each peer educator group reporting each outcome, and compared the proportions across study arms using a linear regression model with a term for study arm and for site. This model avoids the need to model the covariance structure by analyzing at the unit of randomization (the peer educator group). We also compared the effect of HIVST either via delivery or coupon versus standard testing on HIV testing and linkage to care outcomes by pooling participants in the delivery and coupon arms in a non-prespecified secondary analysis.

Data collection for the 1-month study assessments was interrupted after approximately 85% of participants had completed their assessment, and was delayed for approximately 1 month due to logistical issues with study operations. Participants who were interviewed late who tested during the first month of the study therefore would have responded that their most recent test was more than 1 month ago. All participants were included in our primary analysis per our prespecified analysis plan. As a non-prespecified sensitivity analysis, we assessed HIV testing in the previous 3 months as measured at the 1-month visit. Given that participants were not eligible to participate if they had tested in the 3 months prior to enrollment, past 3-month testing captures recent testing during the study for all participants. An additional sensitivity analysis was run for the primary analysis (HIV testing within the past month) with a term for whether the participant was assessed before or after the delay.

Our prespecified primary analysis was a complete-case analysis. Analyses were intention-to-treat, with the exception of linkage to care and ART initiation outcomes, which were conditioned on self-reported HIV status. All tests were 2-sided with no adjustments for multiple comparisons. All analyses were conducted in Stata 14.1 (StataCorp, College Station, TX).

## Results

**Fig 1:**
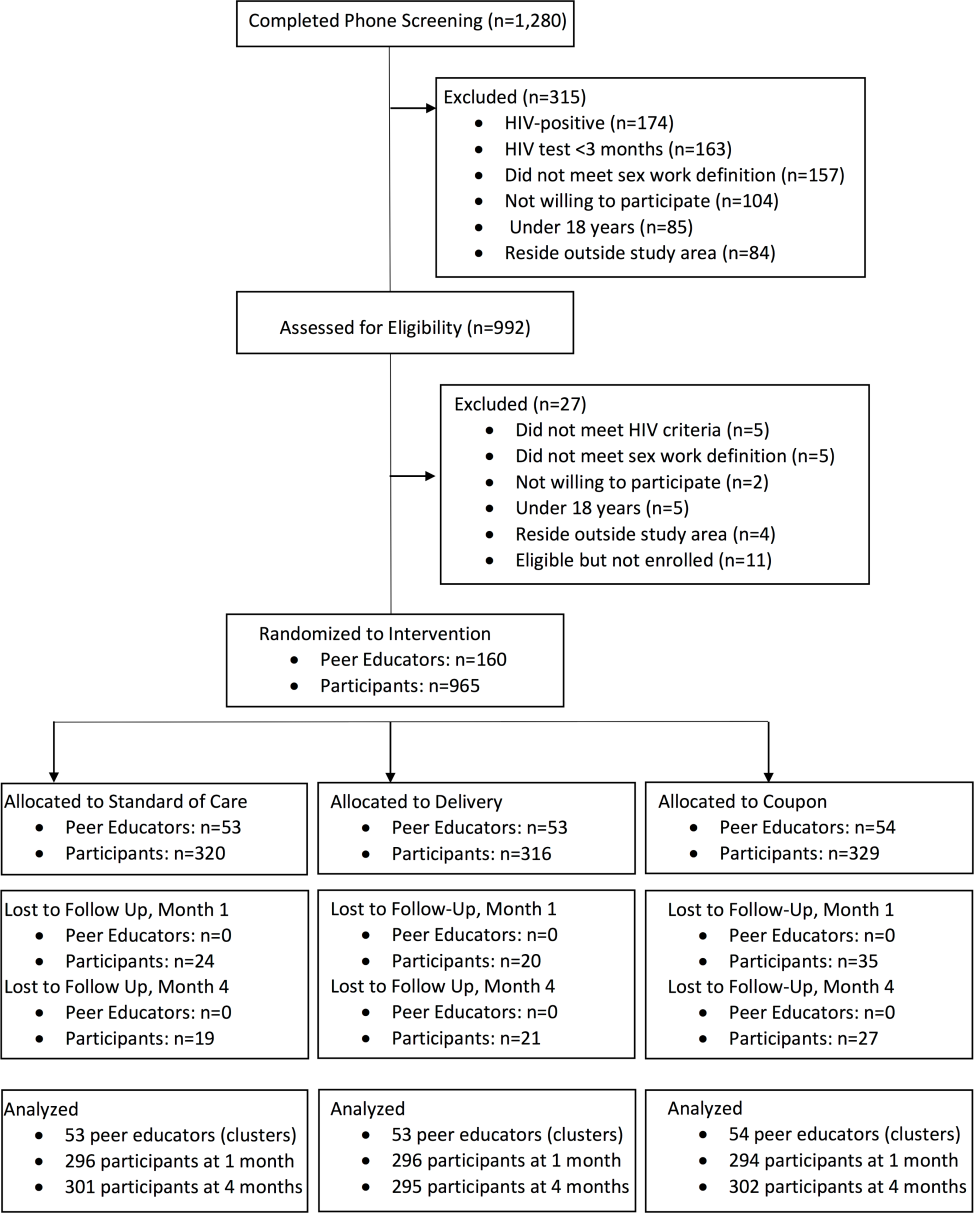
CONSORT flow diagram of screened, randomized, and analyzed participants.

Between September 16 and October 12, 2016, 1,280 potential participants were screened and 965 were eligible and enrolled in the study (Fig 1); 160 peer educator–participant groups were randomized to 1 of 3 study arms (mean 6.0 participants per peer educator group, range 4 to 8). Baseline characteristics were similar between the 3 groups (Table 1). A total of 885 (91.7%) of participants returned for follow-up at 1 month, and 898 (93.1%) returned at 4 months, which comprised the analytic population. There was no difference by study arm in follow-up at 1 month (*P =* 0.35) or 4 months (*P =* 0.65).

**Table 1:** Baseline descriptive characteristics by randomization arm.

Characteristic	Standard-of-care testing (*N =* 320)	Direct HIV self-test delivery (*N =* 316)	HIV self-test coupon (*N =* 329)
**Age, years (median, IQR)**	25 (22 to 31)	25 (21 to 30)	25 (21 to 30)
**Site**			
Livingstone	156 (48.8%)	162 (51.3%)	162 (49.2%)
Kapiri Mposhi	87 (27.2%)	76 (24.1%)	82 (24.9%)
Chirundu	77 (24.1%)	78 (24.7%)	85 (25.8%)
**Have a primary partner**	203 (63.6%)	171 (54.1%)	202 (61.0%)
**Can read and write**	226 (70.9%)	243 (77.1%)	253 (77.9%)
**Education**			
No formal education	53 (16.6%)	30 (9.5%)	25 (7.5%)
Primary/junior	129 (40.3%)	152 (48.1%)	169 (51.5%)
Secondary	131 (40.9%)	128 (40.5%)	130 (39.6%)
Vocational	6 (1.9%)	6 (1.9%)	1 (0.3%)
Tertiary	1 (0.3%)	0	3 (0.9%)
**Mobile phone ownership**	271 (84.7%)	265 (83.9%)	284 (86.3%)
**Monthly income**			
No income	81 (25.8%)	58 (18.7%)	63 (19.4%)
<250 kwacha^1^	40 (12.7%)	32 (10.3%)	51 (15.7%)
251–500 kwacha^1^	75 (23.9%)	86 (27.7%)	74 (22.8%)
501–1,000 kwacha^1^	74 (23.6%)	82 (26.4%)	90 (27.8%)
1,001–1,500 kwacha^1^	17 (5.4%)	30 (9.7%)	26 (8.0%)
>1,500 kwacha^1^	27 (8.6%)	23 (7.4%)	20 (6.2%)
**Years in sex work (median, IQR)**	5 (3 to 10)	5 (3 to 10)	5 (3 to 8)
**Inconsistent condom use with clients**	231 (75.2%)	236 (78.7%)	228 (71.0%)
**Timing of last HIV test**			
>3–6 months	131 (42.3%)	94 (29.8%)	152 (47.1%)
>6–12 months	69 (22.3%)	95 (30.2%)	76 (23.5%)
>12–24 months	18 (5.8%)	26 (8.3%)	26 (8.1%)
>24 months	17 (5.5%)	24 (7.6%)	24 (7.4%)
Never tested	75 (24.2%)	76 (24.1%)	45 (13.9%)
**Intimate partner violence, past 12 months**			
Physical	165 (51.6%)	150 (50.8%)	168 (51.1%)
Sexual	148 (46.4%)	157 (49.7%)	144 (43.8%)
Any	196 (61.4%)	194 (61.4%)	199 (60.5%)

Data given as number (percent) unless otherwise indicated.

^1^50 kwacha = approximately US$1.

### One-month HIV testing

Overall, 89.3% and 79.6% of participants reported testing for HIV (all types of testing) in the previous 1 month at the 1- and 4-month assessment, respectively. At 1 month, 94.9% and 84.4% of participants in the delivery and coupon arms reported testing in the past month, compared to 88.5% in the standard-of-care arm (Table 2). The differences between the HIVST arms and the standard of care arm were not statistically significant (RR delivery versus standard of care 1.07, 95% CI 0.99–1.15, *P =* 0.10; RR coupon versus standard of care 0.95, 95% CI 0.86–1.05, *P =* 0.29). Participants in the delivery arm were statistically significantly more likely to report testing in the past 1 month than participants in the coupon arm (RR 1.13, 95% CI 1.04–1.22, *P =* 0.005; S3 Table). The observed intracluster correlation coefficient for the 1-month primary outcome was 0.58 (95% CI 0.42–0.72). The results for past 1-month HIV testing at the 1-month visit were robust to a sensitivity analysis including a term for whether the participant was assessed before or after the delay in outcome assessment (RR delivery versus standard of care 1.05, 95% CI 0.98–1.13, *P =* 0.19; RR coupon versus standard of care 0.97, 95% CI 0.88–1.06, *P =* 0.44; RR delivery versus coupon 1.09, 95% CI 1.00–1.18, *P =* 0.045).

**Table 2:** HIV testing and linkage to care at 1 and 4 months by study arm.

Outcome	One month				Four months			
	Standard of care (*N =* 296)	Delivery (*N =* 296)	Coupon (*N =* 294)	*P* value	Standard of care (*N =* 301)	Delivery (*N =* 295)	Coupon (*N =* 302)	*P* value
**Tested for HIV in past 1 month**	262 (88.5%)	280 (94.9%)	248 (84.4%)	0.10^1^ 0.29^2^	226 (75.1%)	248 (84.1%)	241 (79.8%)	0.11^1^ 0.42^2^
**Tested for HIV in past 3 months**^**3**^	290 (98.0%)	288 (97.6%)	271 (92.2%)	0.83^1^ 0.01^2^	n/a	n/a	n/a	
**Last HIV test was facility-based**	275 (93.2%)	19 (6.5%)	49 (16.9%)	<0.001^1^ <0.001^2^	282 (93.7%)	13 (4.4%)	33 (11.0%)	<0.001^1^ <0.001^2^
**Tested at least once during study period**	n/a	n/a	n/a	n/a	286 (96.6%)	287 (99.7%)	277 (96.5%)	0.12^1^ 0.92^2^
**HIV status at last test**								0.59 0.60
Positive	59 (20.5%)	49 (16.7%)	36 (12.4%)	0.24	84 (28.2%)	74 (25.3%)	77 (25.7%)	
Negative	194 (67.4%)	222 (75.5%)	217 (74.6%)	0.04	203 (68.1%)	208 (71.2%)	214 (71.3%)	
Unsure	1 (0.4%)	6 (2.0%)	5 (1.7%)		3 (1.0%)	2 (0.7%)	2 (0.7%)	
Inconclusive	0	3 (1.0%)	4 (1.4%)		0	1 (0.3%)	1 (0.3%)	
Prefer not to answer	34 (11.8%)	14 (4.8%)	29 (10.0%)		8 (2.7%)	7 (2.4%)	6 (2.0%)	
**Linked to care (among those testing positive)**	44 (74.6%)	25 (51.0%)	19 (52.8%)	0.07^1^ 0.12^2^	72 (85.7%)	53 (71.6%)	59 (76.6%)	0.13^1^ 0.17^2^
**On ART (among those testing positive)**	27 (46.6%)	11 (22.5%)	9 (25.0%)	0.09^1^ 0.21^2^	54 (64.3%)	35 (48.0%)	44 (57.1%)	0.17^1^ 0.39^2^
**Aware of HIV status**^**4**^	n/a	n/a	n/a	n/a	192 (86.9%)	222 (90.2%)	194 (90.2%)	0.30^1^ 0.30^2^

Data given as number (percent).

^1^*P*-value for delivery arm versus standard arm.

^2^*P*-value for coupon arm versus standard arm.

^3^Due to an interruption in data collection for the 1-month visits, some visits were conducted >1 month after the first peer educator visit, and thus some participants reported that they had not had an HIV test in the past month but they had had an HIV test since their peer educator visit. Note that past 1-month HIV testing is the prespecified primary outcome.

^4^*N =* 682 due to non-participation in the assessment, measured via asking participant to report current HIV status and confirming with a rapid test.

n/a, not applicable.

### Four-month HIV testing

At 4 months, 84.1% of participants in the delivery arm and 79.8% in the coupon arm reported past 1-month testing (all types of tests), compared to 75.1% in the standard-of-care arm (Table 2). There was no significant difference in past-month HIV testing between the delivery (RR 1.11, 95% CI 0.98–1.27, *P =* 0.11) or coupon (RR 1.06, 95% CI 0.92–1.22, *P =* 0.42) arm compared to the standard-of-care arm. There was also no difference in past-month HIV testing at 4 months between the delivery and coupon arms (RR 1.05, 95% CI 0.94–1.18, *P =* 0.40; S3 Table). In all, 80.4% of women who reported at the 1-month visit that their most recent HIV test was positive reported testing for HIV at 4 months compared to 84.9% of women who reported at 1 month that their most recent HIV test was negative.

Participants at both 1 and 4 months in the HIVST arms were significantly less likely to test for HIV in a facility than participants in the standard-of-care arm (Table 2). There was no difference across study arms in testing at least once during the study period (Table 2).

### Linkage to care

At 1 month, 144 (16.3%) participants reported that their most recent HIV test was positive, which increased to 235 (26.2%) at 4 months. At 1 month, among women reporting that their most recent HIV test was positive, 61.1% reported that they had sought medical care. Nearly three-quarters (74.6%) of participants in the standard-of-care arm reported they had sought care following their positive test, compared to 51.0% in the delivery arm and 52.8% in the coupon arm (Table 2). These differences were not statistically significant, although in a non-prespecified analysis pooling the HIVST arms, participants testing positive in the self-testing arms were 27% less likely to seek care for their HIV than participants in the standard-of-care arm (RR 0.73, 95% CI 0.56–0.96, *P =* 0.03). At 1 month, 46.6% of participants who had tested positive in the standard-of-care arm reported initiating ART, compared to 22.5% and 25.0% in the delivery and coupon arms, respectively. These differences were not statistically significant, although there was a non-significant 42% lower proportion of ART initiation in the HIVST arms compared to the standard-of-care arm (RR 0.58, 95% CI 0.33–1.00, *P =* 0.05).

**Fig 2:**
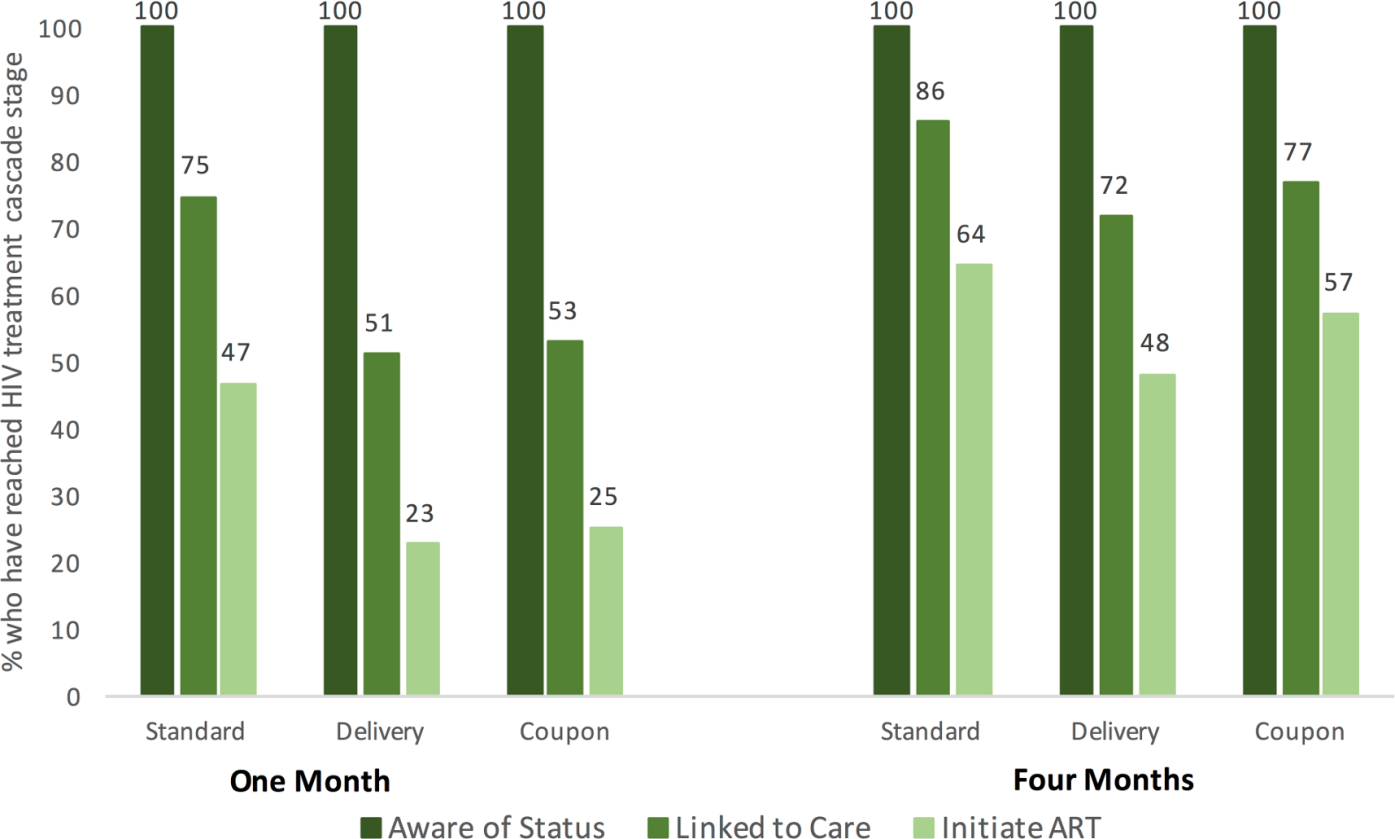
HIV care cascade among women who self-reported a positive HIV status at 1 and 4 months after randomization. Note that by definition all women are aware of their status, as this figure is restricted to women who self-reported having tested positive for HIV.

Linkage to care and ART initiation increased in all arms by 4 months (Fig 2). Of those who had tested positive by 4 months, 85.7% in the standard-of-care arm, 71.6% in the delivery arm, and 76.6% in the coupon arm reported linking to care. Approximately half of participants in the delivery and coupon arms (48.0% and 57.1%, respectively) reported initiating ART by 4 months, compared to 64.3% in the standard-of-care arm. There were no significant differences in linkage to care or ART initiation at 4 months by study arm.

### HIV status knowledge

A total of 682 women (75.9% of those returning for 4-month follow-up) participated in the HIV status knowledge assessment. Of these women, 223 (32.7%) tested positive with a rapid test. Of the women testing positive, 156 (70.0%) self-reported that they were positive. Of 459 women testing negative, 452 (98.5%) reported a negative status. Overall, 89.2% of individuals correctly identified their status. There was no difference in HIV status knowledge between the 3 arms (Table 2).

### HIV self-test use

In the HIVST arms, 92.3% and 89.5% of participants reported using the HIV self-test at 1 and 4 months, respectively. At 1 month, participants in the delivery arm were more likely to report using the HIV self-test compared to participants in the coupon arm (RR delivery versus coupon 1.14, 95% CI 1.05–1.23, *P =* 0.001). There was no significant difference in HIV self-test use at 4 months (RR delivery versus coupon 1.01, 95% CI 0.93–1.09, *P =* 0.88; Table 3). At 4 months, 84.3% of participants in the delivery arm reported using 2 self-test kits, and 83.2% in the coupon arm reported using 2 kits (RR delivery versus coupon 1.02, 95% CI 0.92–1.12, *P =* 0.75). Overall, at least 1 HIV self-test was collected from 14.2% of participants, 17.7% in the delivery arm and 12.2% in the coupon arm (RR delivery versus coupon 1.58, 95% CI 0.56–4.45, *P =* 0.38). One self-test was collected from 47.7% of participants who reported using 1 test kit over the course of the study. No test kits were collected from participants who reported using no test kits over the course of the study, and at least 1 test kit was collected from 9.2% of participants who reported using 2 test kits over the course of the study.

**Table 3:** HIV self-test kit distribution and use at 1 and 4 months by study arm.

Outcome	One month			Four months		
	Delivery (*N =* 289)	Coupon (*N =* 285)	*P* value^1^	Delivery (*N =* 295)	Coupon (*N =* 299)	*P* value^1^
**Offered coupon/test by peer educator**	285 (98.6%)	273 (95.5%)	0.17	284 (96.3%)	293 (98.0%)	0.20
**Took coupon/test from peer educator**	285 (98.6%)	272 (95.1%)	0.17	284 (96.3%)	291 (97.3%)	0.52
**Received HIV self-test**^**2**^	285 (100%)	258 (90.2%)	0.003	284 (100%)	280 (93.7%)	0.003
**Used HIV self-test**	284 (98.3%)	246 (86.3%)	0.001	265 (89.8%)	266 (89.3%)	0.88
**Number of kits used during study**	n/a	n/a	n/a			0.75
0				0	4 (1.4%)	
1				45 (15.4%)	44 (15.4%)	
2				246 (84.3%)	238 (83.2%)	
**Number of tests returned**^**3**^	n/a	n/a	n/a			0.38
0				224 (84.4%)	231 (87.8%)	
1				24 (8.8%)	18 (6.8%)	
2				24 (8.8%)	14 (5.3%)	

Data given as number (percent).

^1^Multilevel mixed-effects generalized linear model with study arm and site as fixed effects and peer educator group as a random effect.

^2^All participants in the delivery arm received a self-test by definition; in coupon arm, “received” indicates they collected the HIV self-test.

^3^Measured via incentivized collection at the end of the study.

n/a, not applicable.

### Adverse events

Four instances of intimate partner violence related to study participation were reported, 2 in the delivery arm and 2 in the coupon arm. Three participants reported physical violence following their primary partner learning of their HIV self-test use, and 1 reported physical and sexual violence following her primary partner learning about her engagement in sex work. One death was reported in the delivery arm, which was not related to study participation. No other adverse events were reported.

## Discussion

In this study of FSWs in Zambia, a majority of participants at 1- and 4-month assessments reported use of HIV self-tests that were provided either directly via a peer educator or via health clinics or pharmacies. At the 1-month visit, more participants who directly received an HIV self-test reported using the self-test compared to those who had to collect the test from a health facility, and more participants in the delivery arm reported any HIV testing in the previous month compared to the coupon arm. However, there was no difference between the HIVST arms in either measure by the 4-month time point. In the short term, direct delivery of the HIV self-test may be more effective because it removes some barriers to using the self-test—such as concerns related to confidentiality or logistical barriers—that are mitigated over time. In many health systems, the most realistic distribution mechanism for HIV self-tests will be via existing health clinics and pharmacies. Furthermore, in the delivery arm, the test is immediately available, whereas in the coupon arm, there is necessarily a delay in collecting and using the test since the coupon requires participants to take time to visit a health facility and collect the HIV self-test. The results of this study indicate that facility provision is acceptable to FSWs and can lead to uptake of HIVST just as high as through direct delivery within a few months.

There was no difference in recent HIV testing coverage among women with access to HIV self-tests compared to women who were referred to existing standard HIV testing facilities, and past 1-month HIV testing coverage exceeded 75% in all study arms. Participants in all arms had access to a peer educator, with whom they met a minimum of 4 times over the course of the study. Previous peer-educator-based interventions in diverse settings have generally led to increased engagement in HIV prevention and care [21–23]. Data from pre-study focus groups with peer educators indicated that stigma at multiple levels is a key barrier to HIV testing among FSWs in Zambian transit towns [17]. The provision of peer educator support in all study arms may have mitigated some concerns related to stigma and may have facilitated HIV testing [21,23–27]. Thus, it is possible that our control arm represented an “augmented” standard of care, leading to HIV testing being higher than we would have seen if we had not worked with peer educators. Nevertheless, peer-educator-based interventions are common among organizations working with FSW populations globally [28,29]. This study indicates that HIV testing interventions (including self-testing) that are delivered via peer educators may have a large effect on HIV testing, supporting their use. Furthermore, the use of an “augmented” standard of care in this study means that the effects shown are likely to be conservative, and may represent an underestimate of the effect of self-test provision.

ART initiation was lower in the HIVST arms compared to the standard-of-care arm, although it approximately doubled between the 1- and 4-month visits, from 25% to 50%. Both linkage to care and ART initiation increased rapidly in the HIVST arms, approaching the standard-of-care arm by 4 months, supporting the hypothesis that linkage to care and ART initiation take longer with HIVST, and mitigating some concern that individuals who self-test will not link to care [30]. By 4 months, ART initiation in the standard testing arm approached previously described estimates of ART coverage among FSWs in Zimbabwe [14] and exceeded a previous global estimate of 36% among FSWs in low- and middle-income countries [31]. In the general population in Zambia, 65% of people living with HIV are on ART [1]. In this study, where participants had access to a peer educator, linkage to care and ART initiation following a positive HIV self-test was high and increased over time. Future studies should consider linkage to care and ART interventions following HIVST, including the role of peer educators for facilitating HIV care cascade progression.

There were 3 cases of intimate partner violence related to HIVST in this study. We monitored intimate partner violence throughout the study as a potential adverse event related to HIVST. Intimate partner violence is a concern in HIVST interventions where participants test with or distribute kits to partners [12,32]. In this study, participants were only given HIV self-test kits for their own use. However, intimate partner violence is common among FSWs, and it is possible that personal use of HIVST could lead to intimate partner violence if, for example, a partner found the HIV self-test kit. We noted a lower rate of intimate partner violence related to HIVST in this study compared to a previous study of HIV self-test provision for partner testing among FSWs in Kenya (0.3% in the current study compared to 2% in the Kenya study) [12]. This lower rate may be at least in part due to the fact that we did not include partner testing in this study. These results indicate that HIVST among FSWs is safe when provided for their own use, although the potential for intimate partner violence following HIVST should be considered, and resources for women experiencing violence made available.

The results of this study must be considered in the context of several limitations. The majority of outcomes in this study relied on self-report, including HIV testing, linkage to care, and ART initiation outcomes. It is possible that participants were affected by social desirability bias, which likely would have led to overestimation of HIV testing and underestimation of positive HIV test results, which could affect answers to questions related to progression in the HIV care cascade. Although we attempted to improve measurement of HIV self-test kit use by buying kits back at the end of the study, this occurred only 1 month after the last kit was distributed, and it is possible that not all kits that would have been used eventually had been used by then. Participants in all 3 arms were recruited in the same study communities, and thus there could have been contamination between arms. We attempted to minimize this by distributing only a single HIV self-test kit at a time and by requiring a study coupon in exchange for the HIV self-test kit in the coupon arm. Participants who lost their coupon would not be able to collect the test kit; however, report of collection and use at both time points was high. Analyses of linkage to care and ART initiation were restricted to individuals who reported a positive HIV test, and thus do not represent a randomized comparison. In addition, with only 4 months of follow-up, the time frame for linkage to care and ART initiation was short. It is possible that over a longer time period the percentage of participants seeking care and initiating ART in the HIVST arms would approach that of the standard-of-care arm. However, to our knowledge this study represents one of the largest samples of individuals reporting positive HIV status following HIVST reported in the literature, and thus provides important data on HIV care cascade progression following self-testing. The power calculation for the study was based on an estimate that 50% of participants in the standard-of-care arm would test for HIV. Actual HIV testing coverage was much higher, and as such the trial may have been underpowered. However, loss to follow-up was lower than the anticipated 20%. During the 1-month study assessment, there was an unavoidable delay in data collection that may have resulted in an underestimate of past-month HIV testing. This study was conducted among Zambian FSWs in transit towns with relatively little involvement in HIV research. The results of this study may not be generalizable to populations of FSWs that are more engaged in research or in higher socioeconomic brackets. This sample was drawn from the social networks of peer educators, which could further affect generalizability. However, the age, educational attainment, and HIV testing history of our sample were very similar to those of a previous survey of FSWs in the same towns conducted via time-location sampling [18]. These results are therefore likely generalizable to FSWs working in similar contexts.

We demonstrate high uptake of HIVST in a sample of FSWs living in Zambian transit towns. Although women appeared to adopt the tests more quickly when they were directly distributed, by 4 months women who had access to HIV self-tests via a coupon that could be used to collect them from a health clinic or pharmacy used the test kits at a rate similar to that of women who received them directly. These findings suggest that distribution of HIV self-tests via existing health infrastructure, such as pharmacies and clinics, will be acceptable in populations to which this study is generalizable. Linkage to care and ART initiation were relatively high in the HIVST arms, although additional research and monitoring is needed to ensure that the HIV care cascade targets are met following HIVST. Or findings indicate that HIVST is acceptable, accessible, and safe for FSWs in Zambia, and should be considered as part of a national HIV testing strategy.

## Supporting information

S1 CONSORT Checklist CONSORT checklist. (DOC)

S1 Protocol Full study protocol for the Zambian Peer Educators for HIV self-testing (ZEST) study. (PDF)

S1 Table Risk ratios for HIV self-test use: Delivery versus coupon arm. (DOCX)

S2 Table Risk ratios for HIV testing and linkage to care: Delivery and coupon arms versus standard-of-care arm. (DOCX)

S3 Table Risk ratios for HIV testing and linkage to care: Coupon arm versus delivery and standard-of-care arms. (DOCX)

S4 Table HIV testing and linkage to care: HIV self-testing (pooled delivery and coupon arms) versus standard of care. (DOCX)

S5 Table HIV self-test use in models analyzed at the peer educator level: Delivery versus coupon arm. (DOCX)

S6 Table HIV testing and linkage to care in models analyzed at the peer educator level. (DOCX)

S7 Table Peer educator visits by arm. (DOCX)

S8 Table Mixed-effects logistic regression models for primary and secondary outcomes. (DOCX)

S1 Text Zambian Peer Educators for HIV self-testing study institutional review board approval: ERES Converge, Lusaka, Zambia. (PDF)

S2 Text Zambian Peer Educators for HIV self-testing study institutional review board approval: Harvard T.H. Chan School of Public Health, Boston, Massachusetts, US. (PDF)

## Abbreviations

ART: antiretroviral therapy
FSW: female sex worker
HIVST: HIV self-testing
RR: risk ratio
UNAIDS: Joint United Nations Programme on HIV/AIDS
WHO: World Health Organization
ZEST: Zambian Peer Educators for HIV Self-Testing
