# Supplementary material for: HIV self-testing among female sex workers in Zambia: A cluster randomized controlled trial
Source: PLoS Med. 2017 Nov 21;14(11):e1002442. doi: 10.1371/journal.pmed.1002442 (PMC5697803; doi:10.1371/journal.pmed.1002442)
Supplement: S1 Table — (DOCX) [file pmed.1002442.s003.docx]

**S1 Table.** Risk ratios for HIV self-test use, delivery versus coupon arm

|  | **One Month** | | **Four Months** | |
| --- | --- | --- | --- | --- |
|  | **RR (95% CI)** | **P-value** | **RR (95% CI)** | **P-value** |
| Offered coupon/test by peer educator | 1.03 (0.99 to 1.08) | 0.17 | 0.98 (0.95 to 1.01) | 0.20 |
| Took coupon/test from peer educator | 1.04 (0.98 to 1.09) | 0.17 | 0.99 (0.95 to 1.02) | 0.52 |
| Collected test kit | 1.11 (1.04 to 1.19) | 0.003 | 1.07 (1.02 to 1.12) | 0.003 |
| Used HIV self-test | 1.14 (1.05 to 1.23) | 0.001 | 1.01 (0.93 to 1.09) | 0.88 |
| Used HIV self-test, among those who had the kit | 1.04 (1.01 to 1.08) | 0.01 | 0.98 (0.93 to 1.03) | 0.45 |
| Report using both tests | n/a | n/a | 1.02 (0.92 to 1.12) | 0.75 |
| Returned at least one kit | n/a | n/a | 1.58 (0.56 to 4.45) | 0.38 |
