# Supplementary material for: HIV self-testing among female sex workers in Zambia: A cluster randomized controlled trial
Source: PLoS Med. 2017 Nov 21;14(11):e1002442. doi: 10.1371/journal.pmed.1002442 (PMC5697803; doi:10.1371/journal.pmed.1002442)
Supplement: S2 Table — (DOCX) [file pmed.1002442.s004.docx]

**S2 Table.** Risk ratios for HIV testing and linkage to care, delivery and coupon versus standard

|  | **One Month** | | **Four Months** | |
| --- | --- | --- | --- | --- |
|  | **RR (95% CI)** | **P-value** | **RR (95% CI)** | **P-value** |
| Tested for HIV in past one month  Standard-of-Care  Delivery  Coupon | 1.00  1.07 (0.99 to 1.15)  0.95 (0.86 to 1.05) | 0.10  0.29 | 1.00  1.11 (0.98 to 1.27)  1.06 (0.92 to 1.22) | 0.11  0.42 |
| Tested for HIV in past three months  Standard-of-Care  Delivery  Coupon | 1.00  1.00 (0.97 to 1.03)  0.94 (0.90 to 0.99) | 0.83  0.01 | n/a | n/a |
| Last HIV test was facility-based  Standard-of-Care  Delivery  Coupon | 1.00  0.07 (0.03 to 0.14)  0.18 (0.12 to 0.27) | <0.001  <0.001 | 1.00  0.05 (0.02 to 0.10)  0.12 (0.08 to 0.18) | <0.001  <0.001 |
| Tested positive  Standard-of-Care  Delivery  Coupon | 1.00  0.78 (0.51 to 1.20)  0.62 (0.39 to 0.97) | 0.26  0.04 | 1.00  0.91 (0.66 to 1.27)  0.92 (0.65 to 1.28) | 0.59  0.60 |
| Linked to care (among those testing positive)  Standard-of-Care  Delivery  Coupon | 1.00  0.72 (0.51 to 1.02)  0.75 (0.52 to 1.07) | 0.07  0.12 | 1.00  0.86 (0.71 to 1.04)  0.89 (0.76 to 1.05) | 0.13  0.17 |
| On ART  Standard-of-Care  Delivery  Coupon | 1.00  0.55 (0.27 to 1.10)  0.62 (0.30 to 1.30) | 0.09  0.21 | 1.00  0.77 (0.56 to 1.11)  0.88 (0.66 to 1.18) | 0.17  0.39 |
| Correctly identified HIV status  Standard-of-Care  Delivery  Coupon | n/a | n/a | 1.00  1.04 (0.97 to 1.11)  1.04 (0.97 to 1.11) | 0.30  0.30 |
