# Supplementary material for: HIV self-testing among female sex workers in Zambia: A cluster randomized controlled trial
Source: PLoS Med. 2017 Nov 21;14(11):e1002442. doi: 10.1371/journal.pmed.1002442 (PMC5697803; doi:10.1371/journal.pmed.1002442)
Supplement: S3 Table — (DOCX) [file pmed.1002442.s005.docx]

**S3 Table.** Risk ratios for HIV testing and linkage to care, coupon versus delivery and standard

|  | **One Month** | | **Four Months** | |
| --- | --- | --- | --- | --- |
|  | **RR (95% CI)** | **P-value** | **RR (95% CI)** | **P-value** |
| Tested for HIV in past one month  Coupon  Delivery  Standard | 1.00  1.13 (1.04 to 1.22)  1.05 (0.96 to 1.17) | 0.005  0.29 | 1.00  1.05 (0.94 to 1.18)  0.94 (0.82 to 1.09) | 0.40  0.42 |
| Tested for HIV in past three months  Coupon  Delivery  Standard | 1.00  1.06 (1.01 to 1.12)  1.06 (1.01 to 1.12) | 0.03  0.01 | n/a | n/a |
| Last HIV test was facility-based  Coupon  Delivery  Standard | 1.00  0.38 (0.17 to 0.88)  5.47 (3.64 to 8.23) | 0.02  <0.001 | 1.00  0.40 (0.18 to 0.93)  8.44 (5.45 to 13.1) | 0.03  <0.001 |
| Tested positive  Coupon  Delivery  Standard | 1.00  1.27 (0.80 to 2.01)  1.62 (1.03 to 2.56) | 0.31  0.04 | 1.00  1.00 (0.72 to 1.39)  1.09 (0.78 to 1.53) | >0.99  0.60 |
| Linked to care (among those testing positive)  Coupon  Delivery  Standard | 1.00  0.96 (0.61 to 1.51)  1.34 (0.93 to 1.92) | 0.87  0.12 | 1.00  0.97 (0.77 to 1.21)  1.12 (0.95 to 1.31) | 0.76  0.17 |
| On ART  Coupon  Delivery  Standard | 1.00  0.88 (0.35 to 2.20)  1.61 (0.77 to 3.38) | 0.79  0.21 | 1.00  0.89 (0.61 to 1.31)  1.13 (0.85 to 1.52) | 0.56  0.39 |
| Correctly identified HIV status  Coupon  Delivery  Standard | n/a | n/a | 1.00  1.00 (0.94 to 1.06)  0.96 (0.90 to 1.03) | 0.99  0.30 |
