# Supplementary material for: HIV self-testing among female sex workers in Zambia: A cluster randomized controlled trial
Source: PLoS Med. 2017 Nov 21;14(11):e1002442. doi: 10.1371/journal.pmed.1002442 (PMC5697803; doi:10.1371/journal.pmed.1002442)
Supplement: S4 Table — (DOCX) [file pmed.1002442.s006.docx]

**S4 Table.** HIV testing and linkage to care, HIV self-testing (pooled delivery and coupon) versus standard-of-care

|  | **One Month** | | **Four Months** | |
| --- | --- | --- | --- | --- |
|  | **RR (95% CI)** | **P-value** | **RR (95% CI)** | **P-value** |
| Tested for HIV in past one month | 1.01 (0.93 to 1.09) | 0.84 | 1.09 (0.96 to 1.23) | 0.19 |
| Tested for HIV in past three months | 0.97 (0.94 to 1.00) | 0.04 | n/a | n/a |
| Tested positive | 0.70 (0.48 to 1.02) | 0.07 | 0.91 (0.69 to 1.22) | 0.54 |
| Linked to care (among those testing positive) | 0.73 (0.56 to 0.96) | 0.03 | 0.88 (0.77 to 1.00) | 0.06 |
| On ART | 0.58 (0.33 to 1.00) | 0.05 | 0.84 (0.65 to 1.08) | 0.16 |
| Correctly identified HIV status | n/a | n/a | 1.04 (0.97 to 1.11) | 0.25 |
