# Supplementary material for: HIV self-testing among female sex workers in Zambia: A cluster randomized controlled trial
Source: PLoS Med. 2017 Nov 21;14(11):e1002442. doi: 10.1371/journal.pmed.1002442 (PMC5697803; doi:10.1371/journal.pmed.1002442)
Supplement: S5 Table — (DOCX) [file pmed.1002442.s007.docx]

**S5 Table.** HIV self-test use in models analyzed at the peer educator level, delivery versus coupon

|  | **One Month** | | **Four Months** | |
| --- | --- | --- | --- | --- |
|  | **Mean Difference in Proportion (95% CI)** | **P-value** | **Mean Difference in Proportion (95% CI)** | **P-value** |
| Offered coupon/test by peer educator | 0.04 (-0.01 to 0.09) | 0.15 | -0.02 (-0.05 to 0.01) | 0.22 |
| Took coupon/test from peer educator | 0.04 (-0.01 to 0.09) | 0.14 | -0.01 (-0.05 to 0.02) | 0.52 |
| Collected test kit | 0.10 (0.04 to 0.16) | 0.003 | 0.07 (0.02 to 0.11) | 0.004 |
| Used HIV self-test | 0.13 (0.06 to 0.20) | 0.001 | 0.01 (-0.07 to 0.08) | 0.84 |
| Report using both tests | n/a | n/a | 0.04 (-0.05 to 0.14) | 0.34 |
| Returned at least one kit | n/a | n/a | 0.04 (-0.08 to 0.17) | 0.51 |

Estimated with linear regression models with the proportion of each peer educator group reporting each outcome as the outcome and a term for study arm and study site (Kapiri, Chirundu, or Livingstone)
