# Supplementary material for: HIV self-testing among female sex workers in Zambia: A cluster randomized controlled trial
Source: PLoS Med. 2017 Nov 21;14(11):e1002442. doi: 10.1371/journal.pmed.1002442 (PMC5697803; doi:10.1371/journal.pmed.1002442)
Supplement: S6 Table — (DOCX) [file pmed.1002442.s008.docx]

**S6 Table.** HIV testing and linkage to care in models analyzed at the peer educator level

|  | **One Month** | | **Four Months** | |
| --- | --- | --- | --- | --- |
|  | **Mean Difference in Proportion (95% CI)** | **P-value** | **Mean Difference in Proportion**  **(95% CI)** | **P-value** |
| Tested for HIV in past one month  Standard-of-Care  Delivery  Coupon | Ref  0.07 (-0.006 to 0.15)  -0.04 (-0.12 to 0.03) | 0.07  0.27 | Ref  0.09 (-0.02 to 0.20)  0.03 (-0.07 to 0.14) | 0.10  0.52 |
| Tested for HIV in past three months**  Standard-of-Care  Delivery  Coupon | Ref  0.0002 (-0.04 to 0.04)  -0.06 (-0.11 to -0.02) | 0.99  0.005 | n/a | n/a |
| Tested positive  Standard-of-Care  Delivery  Coupon | Ref  -0.07 (-0.15 to 0.02)  -0.11 (-0.20 to -0.02) | 0.15  0.01 | Ref  -0.03 (-0.12 to 0.06)  -0.02 (-0.11 to 0.07) | 0.55  0.69 |
| Linked to care (among those testing positive)  Standard-of-Care  Delivery  Coupon | Ref  -0.27 (-0.49 to 0.05)  -0.22 (-0.45 to 0.01) | 0.02  0.06 | Ref  -0.13 (-0.30 to 0.04)  -0.10 (-0.27 to 0.06) | 0.13  0.22 |
| On ART  Standard-of-Care  Delivery  Coupon | Ref  -0.21 (-0.40 to -0.006)  -0.11 (-0.31 to 0.10) | 0.04  0.31 | -0.13 (-0.33 to 0.06)  -0.08 (-0.27 to 0.11) | 0.19  0.41 |
| Correctly identified HIV status^1^  Standard-of-Care  Delivery  Coupon | n/a | n/a | Ref  0.005 (-0.06 to 0.07)  0.03 (-0.04 to 0.10) | 0.89  0.42 |

Estimated with linear regression models with the proportion of each peer educator group reporting each outcome as the outcome and a term for study arm and study site (Kapiri, Chirundu, or Livingstone)
