# Supplementary material for: HIV self-testing among female sex workers in Zambia: A cluster randomized controlled trial
Source: PLoS Med. 2017 Nov 21;14(11):e1002442. doi: 10.1371/journal.pmed.1002442 (PMC5697803; doi:10.1371/journal.pmed.1002442)
Supplement: S7 Table — (DOCX) [file pmed.1002442.s009.docx]

**S7 Table.** Peer educator visits by arm

|  | **One Month** | | **Four Months** | |
| --- | --- | --- | --- | --- |
|  | **Median (IQR) or N (%)** | **P-value** | **Median (IQR) or N (%)** | **P-value** |
| No peer educator visits in past month  Standard-of-care  Delivery  Coupon | 2 (0.7%)  1 (0.3%)  9 (3.1%) | 0.81  0.20 | 8 (2.7%)  3 (1.0%)  3 (1.0%) | 0.35  0.36 |
| Number of peer educator visits in past month  Standard-of-care  Delivery  Coupon | 2 (2 to 3)  2 (2 to 3)  2 (2 to 3) | 0.55  0.68 | 3 (2 to 4)  2 (2 to 3)  2 (2 to 3) | 0.15  0.33 |
