# Supplementary material for: HIV self-testing among female sex workers in Zambia: A cluster randomized controlled trial
Source: PLoS Med. 2017 Nov 21;14(11):e1002442. doi: 10.1371/journal.pmed.1002442 (PMC5697803; doi:10.1371/journal.pmed.1002442)
Supplement: S8 Table — (DOCX) [file pmed.1002442.s010.docx]

**S8 Table.** Mixed effects logistic regression models for primary and secondary outcomes

|  | **One Month** | | **Four Months** | |
| --- | --- | --- | --- | --- |
|  | **RR (95% CI)** | **P-value** | **RR (95% CI)** | **P-value** |
| Tested for HIV in past one month  Standard-of-Care  Delivery  Coupon | 1.00  3.71 (0.88 to 15.7)  0.51 (0.16 to 1.60) | 0.08  0.25 | 1.00  2.16 (0.83 to 5.63)  1.29 (0.51 to 3.31) | 0.12  0.59 |
| Last HIV test was facility-based  Standard-of-Care  Delivery  Coupon | 1.00  0.0001 (0.00001 to 0.0009)  0.0006 (0.0001 to 0.004) | <0.001  <0.001 | 1.00  0.0003 (0.00006 to 0.002)  0.001 (0.0003 to 0.004) | <0.001  <0.001 |
| Tested positive  Standard-of-Care  Delivery  Coupon | 1.00  0.71 (0.38 to 1.32)  0.51 (0.27 to 0.97) | 0.28  0.04 | 1.00  0.87 (0.52 to 1.44)  0.88 (0.52 to 1.49) | 0.61  0.64 |
| Linked to care (among those testing positive)  Standard-of-Care  Delivery  Coupon | 1.00  0.29 (0.09 to 0.99)  0.33 (0.09 to 1.17) | 0.05  0.09 | 1.00  0.32 (0.09 to 1.11)  0.40 (0.12 to 1.33) | 0.07  0.14 |
| On ART  Standard-of-Care  Delivery  Coupon | 1.00  0.21 (0.04 to 1.16)  0.34 (0.06 to 1.83) | 0.07  0.21 | 1.00  0.44 (0.14 to 1.33)  0.61 (0.20 to 1.81) | 0.15  0.37 |
| Correctly identified HIV status  Standard-of-Care  Delivery  Coupon | n/a | n/a | 1.00  1.39 (0.75 to 2.58)  1.39 (0.75 to 2.61) | 0.30  0.30 |
