# Supplementary material for: HIV self-testing among female sex workers in Zambia: A cluster randomized controlled trial
Source: PLoS Med. 2017 Nov 21;14(11):e1002442. doi: 10.1371/journal.pmed.1002442 (PMC5697803; doi:10.1371/journal.pmed.1002442)
Supplement: S2 Text — (PDF) [file pmed.1002442.s012.pdf]

HARVARD
Human Research Protection Program

Harvard T.H. Chan School of Public Health
Office of Human Research Administration
90 Smith Street, 3rd Floor
Boston, MA 02120

Notification of Initial Study Approval

May 18, 2016

Till Baernighausen
tbaernig@hsph.harvard.edu

**Protocol Title:**	Zambian Peer Educators for HIV Self-Testing (ZEST) Study
**Principal Investigator:**	Till Baernighausen
**Protocol #:**	IRB16-0383
**Funding Source:**	International Initiative for Impact Evaluation (3ie) (Active)
**Review Date:**	5/11/2016
**STUDY Effective Date:**	5/11/2016
**Expiration Date:**	5/10/2017
**IRB Review Type:**	Full Board
**IRB Review Action:**	Approved

On 5/11/2016, the Institutional Review Board (IRB) of the Harvard T.H. Chan School of Public Health approved this Initial Study. **Please note that the approval for this protocol will lapse on 5/10/2017.**

This approval includes the following:

- Initial Application, IRB16-0383
- IRB Protocol: ZEST Research Protocol 14March2016 (1.0)
- Consent Form: ZEST Participant Consent (1.0)
- Recruitment Materials: Recruitment Script (1.0)
- Study Instrument/Tools: Cost-Effectiveness Forms (1.0)
- Study Instrument/Tools: Orasure HIV self-testing instructions (0.01)
- Study Instrument/Tools: Appendix 1: Quantitative Study Forms (1.0)
- Study Instrument/Tools: Appendix 2: Qualitative Study Forms (1.0)
- Other: ZEST Manual of Operations and Procedures (1.0)

The IRB made the following determinations:

- Risk Determination: Greater than minimal risk
- Research Information Security Level: The research is classified, using Harvard's Data Security Policy, as Level 4 Data.

The IRB requests the following:

- Submit translated study documents as soon as they become available, as well as a signed Translation Attestation Form, via Modification in ESTR. Copies of locally-approved translated versions or a status update must be submitted to the IRB within 45 days of this notification letter.
- Upload the local ethical review board approval letter when it becomes available via Modification in ESTR. Note that no human subjects research activities can occur in the field until local review has been secured.

Please contact me at 617-432-5174 or kninsala@hsph.harvard.edu with any questions.

Sincerely,

Keren-Nicole Insalaco
Sr. IRB Review Specialist
