# Supplementary material for: HIV self-testing among female sex workers in Zambia: A cluster randomized controlled trial
Source: PLoS Med. 2017 Nov 21;14(11):e1002442. doi: 10.1371/journal.pmed.1002442 (PMC5697803; doi:10.1371/journal.pmed.1002442)
Supplement: S1 Text — (PDF) [file pmed.1002442.s011.pdf]

ERES CONVERGE

33 Joseph Mwilwa Road
Rhodes Park, Lusaka
Tel: +260 955 155 633
+260 955 155 634
Cell: +260 966 765 503
Email: eresconverge@yahoo.co.uk

I.R.B. No. 00005948
F.W.A. No. 00011697

15th June, 2016

Ref. No. 2016-May-003

The Principle Investigator
Dr. Michael Mulimansenga Chanda & Dr. Till Barnighaussen
John Snow Inc. Company Limited
P.O. Box RW 51185,
LUSAKA.

Dear Dr`s Mulimansenga & Barnighaussen,

RE: ZAMBIAN PEER EDUCATORS FOR HIV SELF- TESTING (ZEST) STUDY.

Reference is made to your resubmission dated June 9, 2016. The IRB resolved to approve this study and your participation as Principal Investigator for a period of one year.

Review Type	Ordinary	Approval No. **2016-May-003**
Approval and Expiry Date	Approval Date: 15th June, 2016	Expiry Date: 14th June, 2017
Protocol Version and Date	Version-Nil	14th June, 2017
Information Sheet, Consent Forms and Dates	• English.	14th June, 2017
Consent form ID and Date	Version September 17, 2015	14th June, 2017
Recruitment Materials	Nil	14th June, 2017
Other Study Documents	Baseline Quantitative Questionnaire.	14th June, 2017
Number of participants approved for study	-	14th June, 2017

Specific conditions will apply to this approval. As Principal Investigator it is your responsibility to ensure that the contents of this letter are adhered to. If these are not adhered to, the approval may be suspended. Should the study be suspended, study sponsors and other regulatory authorities will be informed.

Conditions of Approval

- No participant may be involved in any study procedure prior to the study approval or after the expiration date.
- All unanticipated or Serious Adverse Events (SAEs) must be reported to the IRB within 5 days.
- All protocol modifications must be IRB approved prior to implementation unless they are intended to reduce risk (but must still be reported for approval). Modifications will include any change of investigator/s or site address.
- All protocol deviations must be reported to the IRB within 5 working days.
- All recruitment materials must be approved by the IRB prior to being used.
- Principal investigators are responsible for initiating Continuing Review proceedings. Documents must be received by the IRB at least 30 days before the expiry date. This is for the purpose of facilitating the review process. Any documents received less than 30 days before expiry will be labelled "late submissions" and will incur a penalty.
- Every 6 (six) months a progress report form supplied by ERES IRB must be filled in and submitted to us.
- ERES Converge IRB does not "stamp" approval letters, consent forms or study documents unless requested for in writing. This is because the approval letter clearly indicates the documents approved by the IRB as well as other elements and conditions of approval.

Should you have any questions regarding anything indicated in this letter, please do not hesitate to get in touch with us at the above indicated address.

On behalf of ERES Converge IRB, we would like to wish you all the success as you carry out your study.

Yours faithfully,
ERES CONVERGE IRB

Dr. E. Munalula-Nkandu
BSc (Hons), MSc, MA Bioethics, PgD R/Ethics, PhD
CHAIRPERSON
